# Metazoans and Intrinsic Apoptosis: An Evolutionary Analysis of the Bcl-2 Family

**DOI:** 10.3390/ijms23073691

**Published:** 2022-03-28

**Authors:** Chathura D. Suraweera, Suresh Banjara, Mark G. Hinds, Marc Kvansakul

**Affiliations:** 1 Department of Biochemistry and Chemistry, La Trobe Institute for Molecular Science, La Trobe University, Bundoora, VIC 3086, Australia; chathura.suraweera@monash.edu (C.D.S.); banjarasuresh@gmail.com (S.B.); 2 Bio21 Molecular Science and Biotechnology Institute, The University of Melbourne, Parkville, VIC 3052, Australia

**Keywords:** apoptosis, Bcl-2, evolution, sequence analysis, structure analysis

## Abstract

The B-cell lymphoma-2 (Bcl-2) family is a group of genes regulating intrinsic apoptosis, a process controlling events such as development, homeostasis and the innate and adaptive immune responses in metazoans. In higher organisms, Bcl-2 proteins coordinate intrinsic apoptosis through their regulation of the integrity of the mitochondrial outer membrane; this function appears to have originated in the basal metazoans. Bcl-2 genes predate the cnidarian-bilaterian split and have been identified in porifera, placozoans and cnidarians but not ctenophores and some nematodes. The Bcl-2 family is composed of two groups of proteins, one with an α-helical Bcl-2 fold that has been identified in porifera, placozoans, cnidarians, and almost all higher bilaterians. The second group of proteins, the BH3-only group, has little sequence conservation and less well-defined structures and is found in cnidarians and most bilaterians, but not porifera or placozoans. Here we examine the evolutionary relationships between Bcl-2 proteins. We show that the structures of the Bcl-2-fold proteins are highly conserved over evolutionary time. Some metazoans such as the urochordate *Oikopleura dioica* have lost all Bcl-2 family members. This gene loss indicates that Bcl-2 regulated apoptosis is not an absolute requirement in metazoans, a finding mirrored in recent gene deletion studies in mice. Sequence analysis suggests that at least some Bcl-2 proteins lack the ability to bind BH3-only antagonists and therefore potentially have other non-apoptotic functions. By examining the foundations of the Bcl-2 regulated apoptosis, functional relationships may be clarified that allow us to understand the role of specific Bcl-2 proteins in evolution and disease.

## 1. Introduction

Intrinsic apoptosis is a form of regulated cell death in metazoans initiated via intracellular stimuli and co-ordinated by B-cell lymphoma-2 (Bcl-2) proteins. Activation of the Bcl-2 family triggers a proteolytic cascade that dismantles cellular components leading to phagocytotic clearance [1]. Predating the bilaterian-cnidarian divergence in metazoan evolution [2], the development of apoptosis probably coincided with the necessity to develop a response to pathogen invasion [3,4]. The critical role of apoptosis in both the innate and adaptive immune responses make it likely this role arose prior to its adaptation for other processes such as development, homeostasis, and the removal of damaged or unwanted cells in higher metazoans which is prerequisite for the development of complex multicellular lifeforms [5]. Bcl-2 genes appear robustly conserved across metazoan genomes and have been reported in the genomes of the basal metazoans porifera (sponges), placozoans, and cnidarians (hydra, anemones, jellyfish, corals) [6].

The origins of the Bcl-2 family are obscure but it has been postulated that acquisition of the proto-Bcl-2 gene was a unique event during metazoan evolution and likely arose from horizontal gene transfer from a symbiont [7]. Though it has been speculated that the Bcl-2 genes were derived from toxins [4], they do not bear significant sequence or structural resemblance to known toxins. Instead, two phylogenetically separate groups constitute the tripartite activity of the Bcl-2 family. One group shares an α-helical Bcl-2 fold and have either pro-survival or pro-apoptotic activity while the other group, the BH3-only proteins, neutralizes the pro-survival proteins and is intrinsically disordered [8,9]. In mammals, a network of mutually antagonizing interactions between pro- and anti-survival Bcl-2 family members [10,11,12] controls mitochondrial outer membrane permeabilization (MOMP) and release of apoptosis-initiating factors from mitochondria [8,13]. Distinguishing the Bcl-2 proteins is the presence of conserved sequence motifs, known as Bcl-2 homology (BH) motifs, that form their interaction sites [8,9].

Bcl-2-fold proteins possess up to four BH motifs (BH1-BH4) arranged in the order BH4, BH3, BH1, and BH2 and approximately correspond to helices in the α-helical scaffold. In contrast, the BH3-only proteins bear only the BH3-motif [8,9] and have little sequence conservation outside this region with other Bcl-2 proteins. Ligand binding of the multimotif Bcl-2 proteins with its BH3-ligand is mediated by conserved residues in the ligand BH3 motif and those of the BH1-BH3 motifs on the receptor Bcl-2 protein that form a binding groove [8]. Crucial to the function of Bcl-2 proteins is the presence of a hydrophobic C-terminal transmembrane (TM) region that directs them to the MOM [14,15]. MOM pore formation occurs through oligomerization of the pro-apoptotic Bcl-2-fold proteins (Bax, Bak, or Bok in mammals) [8,13,16]. The basal metazoans including sponges, placozoans, and cnidarians not only bear multiple Bcl-2 family members, but also recognizable BH motifs and a TM region while also sharing mechanistic details of their action with mammals [6].

**Figure 1:**
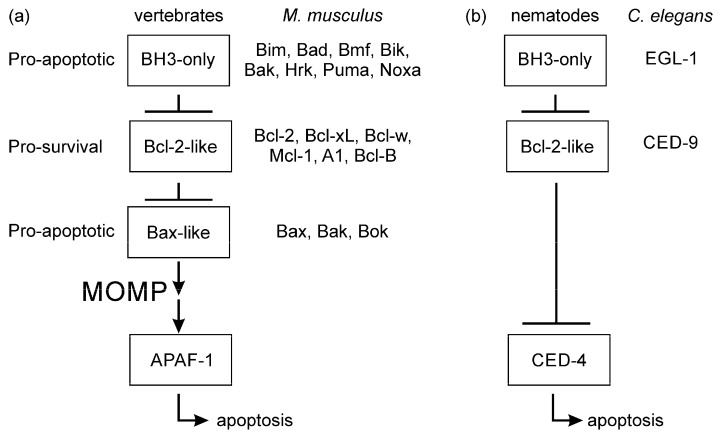
Simplified apoptosis schemes for vertebrates and nematodes. (**a**) The 17 members of the Bcl-2 family of *M. musculus* are indicated in the three classes. (**b**) The Ecdysozoan *C. elegans* apoptosis scheme is much simplified compared to that in mammals bearing a single Bcl-2 protein CED-9 and BH3-only protein EGl-1. The result of either signalling pathway is the activation of caspases that dismantle the cell.

Lineage-specific diversification and gene losses have contributed to numerous differences in apoptotic programs [17], and combined with differences in network size, specialization, specificity, intracellular location, expression levels and cell-type expression establish a complex web of actions for the Bcl-2 proteins. Notwithstanding the conservation of Bcl-2 sequences and structure, evolution has produced mechanistic divergences in their utilization as activators of the caspase cascade [18]. Few detailed studies of apoptosis regulation in invertebrates have been performed. For example, the Ecdysozoans *Caenorhabditis elegans* and *Drosophila melanogaster* have experienced gene loss that has limited the number of Bcl-2 genes present in these organisms. *C. elegans* utilizes a simplified apoptosis initiation mechanism (Figure 1) where the lone Bcl-2 protein (CED-9) binds and inhibits the caspase initiator CED-4 at the MOM. In *C. elegans* the caspase cascade is initiated when the CED-9:CED-4 interaction is antagonized by the BH3-only protein EGL-1 binding CED-9 [19]. This direct scheme of caspase activation is not mirrored in mammals, where nine Bcl-2 proteins and eight BH3-only proteins co-operate in a complex network of interactions to regulate MOMP, a process that does not arise in *C. elegans* apoptosis, to activate the caspases [8,13]. In contrast to the Ecdysozoans, teleost fish such as *Danio rerio* have experienced gene duplication events in their evolutionary history [20], producing an extended number of Bcl-2 family members, and the molecular basis of apoptosis appears similar to those in mammals [21,22]. Recent investigations on the sponge *Geodia cydonium* [23], cnidarian *Hydra vulgaris* [24,25], and the placozoan *Trichoplax adhaerens* [26] indicate that sequences, structures, and interactions parallel those in higher organisms. These studies suggest that not only is there structural conservation, but the mechanism involving interaction of pro- and anti-survival Bcl-2 proteins is conserved with later metazoans.

The foundations of Bcl-2 regulated apoptosis were laid early in metazoan evolution and have largely been maintained throughout their 700-million-year emergence and evolution [27]. Sponges [23], placozoans [26], and cnidarians [24] all preserve the interactions between pro-survival and pro-apoptotic Bcl-2 proteins. BH3-only proteins have been identified in hydra [24] but not in placozoa or porifera. Here we identified Bcl-2 proteins in the genomes of basal metazoans and a surprising complexity in the Bcl-2 family exists in these organisms, for example the sponge *Amphimedon queenslandica* has 7 Bcl-2 proteins [28], the cnidarian *Hydra vulgaris* has at least 11 Bcl-2 family members [29] and the placozoan *Trichoplax adhaerens* has 4 such proteins [30]. In contrast, ctenophores appear to have lost the Bcl-2 family and related intrinsic apoptosis genes, as have a number of other early metazoans. We did not identify any higher organism lacking Bcl-2 family genes, though it is known that certain nematodes lack these genes [31]; a definitive answer is likely to require sampling a broader range of basal metazoan genomes [32]. Understanding the emergence and evolutionary basis of their action may provide insight into the roles of Bcl-2 proteins normal function and disease.

## 2. Results

Database searches for Bcl-2 proteins were performed using sequences of known Bcl-2 folded proteins as seeds. Confirmed Bcl-2 sequences were obtained from *Homo sapiens*, *Mus musculus*, *Gallus gallus*, *Danio rerio*, *C. elegans*, *G. cydonium*, *H. vulgaris,* and *T. adhaerens* (Supplementary Materials). A combination of BLAST [33] and HMM [34] based searches with known Bcl-2 protein as seed sequences were performed to find analogues. Sequences identified as Bcl-2 proteins were subsequently used as seed sequences in an iterative search strategy. In the absence of functional data, in addition to sequence data, naming the homolog was not straightforward where there is low shared sequence identity. We constructed phylogenetic trees performing a bootstrap analysis with 1000 steps as implemented in MEGA7 [35] using the human Bcl-2 proteins. Gene structure and synteny was validated using the NCBI database. The presence of a Bcl-2 fold and the BH motifs were identified by their sequence signatures in the conserved domain database NCBI [36].

### 2.1. Bcl-2 Structures

**Figure 2:**
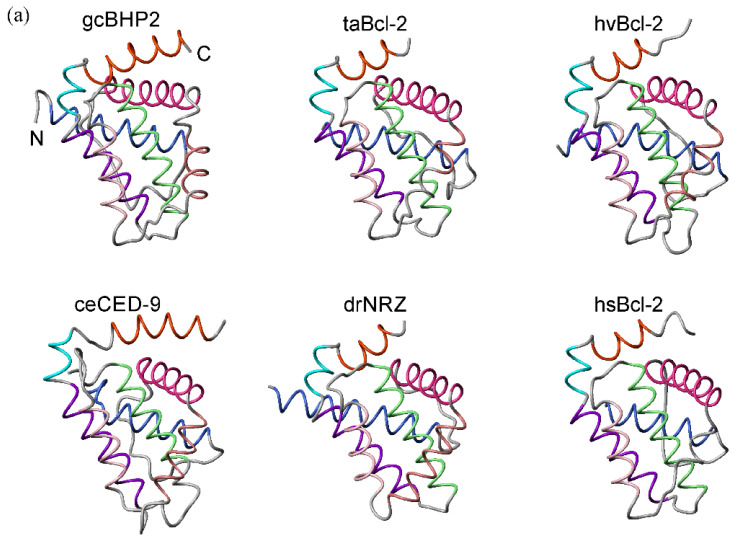

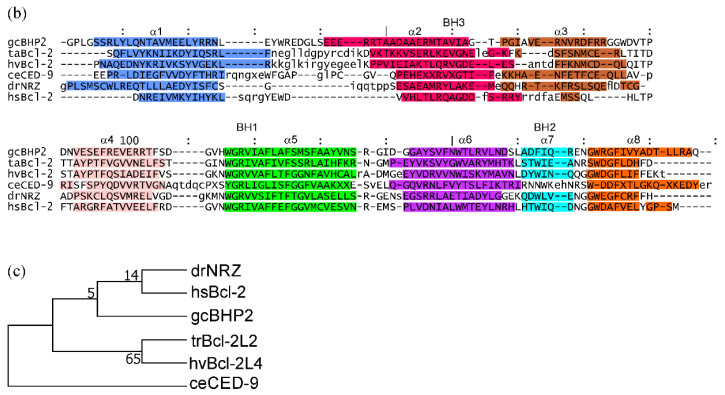
Structural similarity of Bcl-2 proteins derived from early metazoans and bilaterians. The structure of the Bcl-2 fold has been conserved over evolutionary time frames. (**a**) Structurally aligned Bcl-2 proteins. There are relatively minor differences in structure between simple and complex metazoans. Protein data bank (pdb) entries: 5TWA, *G. cydonium* BHP2 (gcBHP2); 6YLD *T adhaerens* Bcl-2L4 (taBcl-2); 6WH0 *H. vulgaris* Bcl-2 (hvBcl2); 1TY4, *C. elegans* CED-9 (ceCED-9); 6FBX *D. rerio* NRZ (drNRZ); 2XA0 *H. Sapiens* Bcl-2 (hsBcl-2). The BH3 ligand of these complexes was removed. Helices are colored: α1 (blue), α2 (deep pink), α3 (brown), α4 (salmon), α5 (green), α6 (purple), α7 (cyan), α8 (orange). Helices are indicated in color according to the alignment. The N and C termini are indicated on gcBHP2 (**b**) Structure based sequence alignment performed using DALI [38] and the PDB files given in (**a**). The helices are indicated by the colored bars (**c**) Dendrogram depicting the relationship between structures based on the structure-sequence alignment of (**b**). The phylogenetic tree was calculated in MEGA7 using the maximum likelihood method and details are provided in the methods and the percentage of trees in which the associated taxa clustered together is shown next to the branches.

The structures of Bcl-2 family proteins from basal metazoans were aligned on their common backbone atoms as determined by distance matrix alignment using DALI [37] (Figure 2a) and the structure-based sequence alignment is shown in Figure 2b and sequence identity and similarity in Table 1. The topology of the Bcl-2-fold has been well-maintained over evolutionary timescales with strikingly similar structures (Figure 2a) but with relatively low shared sequence identities (Table 1). However, there are clear differences too. A structure-guided analysis showed that BHP2 from *G. cydonium* did not cluster with pro-survival Bcl-2 proteins and there were differences in how the BH3 motif was engaged [23]. Structure-based phylogenetic analysis showed the hydra and trichoplax Bcl-2 structures to be the most closely related, while *G. cydonium* BHP2 clustered with those from bilaterians zebra fish and human Bcl-2 while *C. elegans* CED-9 is an outlier (Figure 2c). All Bcl-2 proteins form extensive hydrophobic contacts in the core of the molecule and the BH3 binding groove provided by helices α2–α5 and α8 presents the conserved residues in the BH1-BH3 motifs as a binding surface, as is well-known [8]. Importantly, while there are differences in intermolecular contacts, no structures have provided an alternate binding site for the BH3-ligand. While the structures solved so far provide a limited pool of sequences, it is clear that the overall 3D architecture of the helical bundle is well-preserved from sponge to man with relatively small differences in the core [23]. The major structural differences arise from the presence of unstructured interhelical loops that do not substantially alter the framework of the Bcl-2 helical core. Thus, despite the relatively weak sequence conservation, the core Bcl-2-fold has been well-maintained from the earliest metazoans.

### 2.2. Bcl-2 Genes in Basal Metazoans

The genomes of basal metazoans placozoa (*T. adhaerens*) [30], porifera (the sponges; for example, *Amphimedon queenslandica* [28], *G. cydonium* [40], *Lubomirskia baicalensis* [41]), and cnidaria (for example *H. vulgaris* [29], corals *Acropora millepora* [42], *A. digitifera* [43], sea anemone *Nematostella vectensis* [44], and jellyfish *Aurelia aurita* [45,46]), confirm that these organisms contain Bcl-2 genes (Supplementary Materials). Multiple Bcl-2 paralogs are present in each of these species, with typically two or more multi-motif Bcl-2 genes present. Recognizable Bcl-2 protein sequences were not found in genomes of the ctenophores *Mnemiopsis leidyi* [47] or *Pleurobrachia bachei* [48], however a caspase 7 gene was found in both *M. leidyi* and *P. bachei* indicating apoptosis in these organisms may be initiated by other means. A Blast search of Myxozoans *Thelohanellus kitaue* and *Kudoa iwatai* using the Bcl-2 sequences from *H. vulgaris* did not find related proteins in these organisms, a finding consistent with Panchin et al. [49]. Thus, in both ctenophores and myxozoans the Bcl-2 genes are absent.

**Figure 3:**
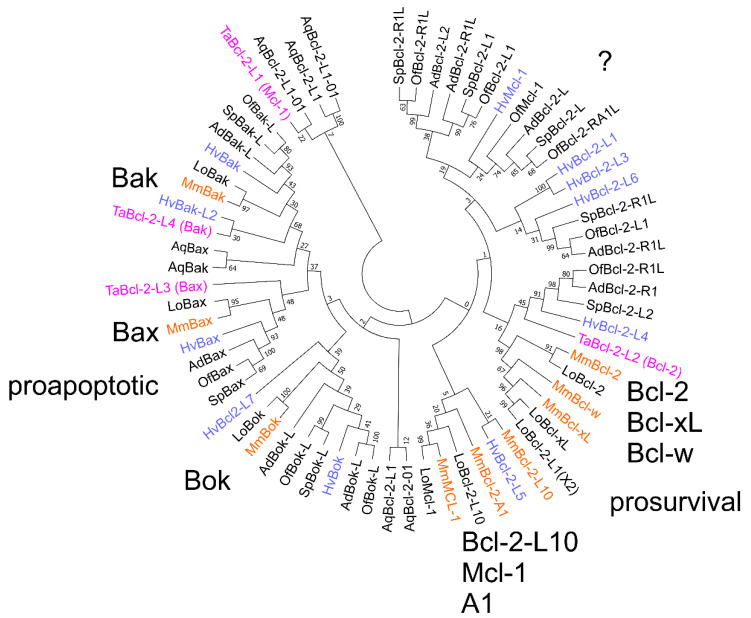
Phylogenetic analysis of Bcl-2 proteins from basal metazoans, garfish and mouse. Evolutionary analyses were conducted in MEGA7 [35] and molecular phylogenetic analysis was performed using the maximum likelihood method as described in the methods section. The analysis involved 66 protein sequences. All positions containing gaps and missing data were eliminated. There were a total of 114 positions in the final dataset and the percentage of trees in which the associated taxa clustered together is shown next to the branches. The mouse Bcl-2 family members are indicated in large letters along with their apoptotic activity. Species abbreviations: Aq, *A. queenslandica*; Mm, *M. musculus*; Hv, *H. vulgaris*; Ta *T. adhaerens*; Ad, *A. digitifera*; Of, *Obicella faveolata*; Sp, *Stylophora pistillata*; Lo, *Lepisosteus oculatus*. Species indicated in colour: *T. adhaerens*, magenta; *H. vulgaris*, blue; *M. musculus*, orange.

A phylogenetic analysis was performed on Bcl-2 sequences from genomes of basal metazoans that are currently available: the sponge *A. queenslandica*, placozoan *T. adhaerens*, cnidarians *H. vulgaris*, *A. digifera*, *Orbicella faveolata*, *Stylophora pistillata,* and the bilaterians *Lepisosteus oculatus* (garfish) and *M. musculus*. As expected, the garfish Bcl-2 family closely matches the mouse Bcl-2 family. Representatives of Bak appeared in all species compared while a Bax homolog appeared absent from *A. queenslandica*. Bok homologs are absent from both the placozaon and *A. queenslandica* genomes. Four multimotif Bcl-2 proteins exist in *T. adhaerens*, Bcl-2L1, Bcl-2L2, Bcl-2L3, and Bcl-2L4 [26,30] in the analysis presented here, Bcl-2L1 and Bcl-2L2 cluster with mouse pro-survival proteins while Bcl-2L3 and Bcl-2L4 cluster with mouse pro-apoptotic proteins Bax and Bak, respectively (Figure 3). Though the results of our analysis vary slightly from those of [26], the conclusion is the same: Bcl-2L1 and Bcl-2L2 cluster with pro-survival proteins. The defining feature of pro-apoptotic proteins, the BH3-motif, is present in Bcl-2L3 and Bcl-2L4, suggesting that, like in mammals, there is a BH3-in-groove interaction for these Bcl-2 proteins. Of note in *T. adhaerens* Bcl-2L4, several residues in the BH1 region are present that would make it unlikely to have a functional BH3-binding groove, as the BH1 motif, RLFISWRRIVTLMAFG, lacks the conserved Gly (NWGR), though other features, BH1, BH3, and BH4, are conserved and the Gly to Arg change potentially signifies an alternative functionality for this protein. The presence of Ser in the equivalent position in the mouse Bcl-2 protein Boo substantially alters the BH3-selectivity compared with the other pro-survival proteins [50]. While sequences from most species cluster well with the pro-apoptotic mouse Bax, Bak, and Bok and the closely related pro-survival proteins Bcl-2, Bcl-x_L_, and Bcl-w, only hydra and garfish have sequences that cluster with the Mcl-1, A1, Bcl-B group. Figure 3 also indicates there is a substantial group of proteins that do not cluster readily with either the pro-survival or pro-apoptotic proteins of bilaterians, including *T. adhaerens* Bcl-2-L1. Though structural and BH3 binding data exist for *G. cydonium* [23], *T. adhaerens* [26], and *H. vulgaris* [25], such data on proteins from other species is limited, however, their conserved sequence features, such as the presence of BH-motifs, conforms to Bcl-2-fold proteins. Nevertheless, further investigations are required to confirm whether members of this cluster perform roles in apoptosis.

In addition to the conservation of sequence motifs Bcl-2 gene structure is also conserved. The genome of the cnidarian *H. vulgaris* contains as many as 11 Bcl-2 family members (9 Bcl-2-like and 2 Bak-like) [28] (Supplementary Materials) and the gene intron/exon structure, at least for the Bak and Bcl-2 members is similar to that for the human genes and indicates they are likely true orthologues of vertebrate Bcl-2 genes [24]. A search of the transcriptome of *Acropora millepora* (Staghorn coral) determined 11 multi-domain Bcl-2 proteins, with a number clustering with specific vertebrate multidomain Bcl-2 proteins, while others were of less certain orthology [51]. Three were assigned to Bok, four assigned to Bcl-w, the other four to Bax, Bak, Mcl-1, and the unrelated Bcl-rambo [51]. The related coral *A. digitifera* has two proteins that cluster with Bok (Figure 3), in addition to single sequences that cluster with mouse Bak, Bax, and Bcl-2, and 4 that are unclassified but are likely pro-survival (Figure 3). In contrast, the sponge *A. queenslandica* has two proteins that cluster with mouse Bak, two with Bcl-2, and 3 unclassified pro-survival proteins (Figure 3). The prevalence of Bok homologues, a relatively poorly studied pro-apoptotic Bcl-2 paralog in mammals, in species as early as cnidarians is striking. Like their mammalian counterparts, the *A. millepora* Bcl-2 proteins were associated with mitochondria through their C-terminal residues, and in the case of Bok, with the ER and Golgi membranes [51].

Conserved intron/exon boundaries exist for the mouse pro-survival Bcl-2 proteins Bcl-2, Bcl-x_L_, Bcl-w, Mcl-1, Bcl-B, and A1 and they generally have a three-exon structure with the first exon untranslated and the coding region formed by splicing exons 2 and 3 [52]. The C-terminal TM region on exon 3 is separate from exon 2 bearing the BH3, BH1 and BH2 motifs (in that order) (Figure 2b). The exon 2/exon 3 boundary is highly conserved with exon 2 ending at a conserved tryptophan in the BH2 motif, GGW. The coding region of *A. queenslandica* Bcl-2-like proteins [28] and *T. adhaerens* [30] Bcl-2L1 are also constructed from two exons with the exon boundary of the first coding exon occurring at the BH2 (GGW) motif. In contrast to the pro-survival Bcl-2 proteins the pro-apoptotic Bax, Bak, and Bok generally have a more complex gene structure consisting of 4–6 exons [52,53]. *A. queenslandica* Bak analogs have four coding exons and the exon boundary at the BH2 GGW motif and TM region is maintained. A key difference of Bok compared to other Bcl-2 proteins in mammals is that the BH3 motif is split between two exons in mammals.

### 2.3. Bilaterian Bcl-2

**Figure 4:**
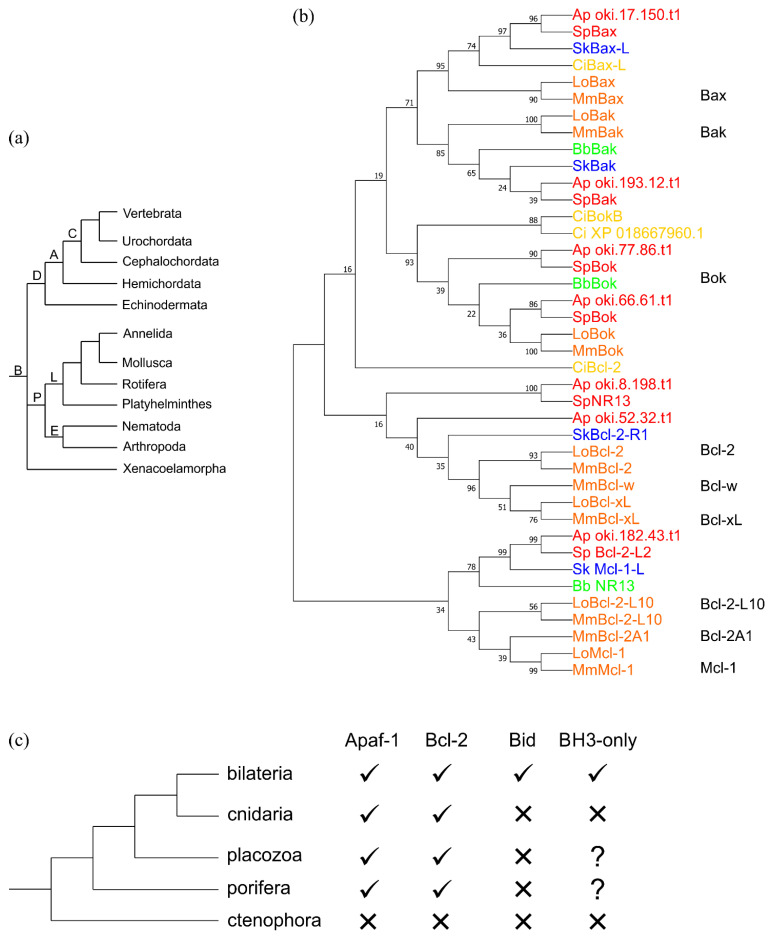
Simplified consensus phylogeny of bilaterians. (**a**) Current bilaterian phylogeny. The branches are indicated: B, Bilaterian; D, Deuterostomia; A, Ambulacraria; C, Chordata; P, Protostomia; L, Lophochotrozoa; E, Ecdysozoa. (**b**) Molecular Phylogenetic analysis by maximum likelihood method of selected metazoans. The mouse Bcl-2 proteins are indicated on the right and the colours signify the phylum; yellow, urochordata *C. intestinalis* (Ci); echinodermata, red, *A. planci*, (Ap); *S. purpuratus* (Sp); blue hemichordata, *S. kowalevskii* (Sk); green Cephalochordata, Bp, *B. belcheri*; orange Vertebrata, *M. musculus*, *L. oculatus*. The evolutionary history was inferred using MEGA7 [34] by the maximum likelihood method and performed on 40 protein sequences having 131 positions in the final dataset. Sequences were aligned in MEGA7 using Muscle and the evolutionary history was inferred by using the maximum likelihood method calculating 1000 replicates and the tree with the highest log likelihood (−10,217.45) is shown. The percentage of trees in which the associated taxa clustered together is shown next to the branches. (**c**) A simplified phylogenetic tree for Bilateria and the basal metazoans indicating the presence or absence of key apoptosis genes. A 🗸 indicates genes(s) are present in the phylum while an ✕ indicates the absence of gene(s).

The Xenocoelomorphs are a basal bilaterian phylum considered the sister group to bilaterians (Figure 4a). The genome of the Xenacoelomorph *Hofstenia miamia* [51], an acoel, lacks identifiable Bcl-2 proteins. In comparison, analysis of genomes from the two main clades of Bilaterians, Protostomia and Deuterostomia [54], show that they too contain multiple Bcl-2 genes. As pointed out above, Protostomes such as the Ecdysozoans, for example *C. elegans* and *D. melanogaster*, have reduced numbers of Bcl-2 family genes compared to basal metazoans, as demonstrated by Nematodes, bearing only a single Bcl-2 protein, while flies have two Bcl-2 genes. However, other Ecdysozoans have multiple Bcl-2 family genes, for example, *Priapulus caudatus*, a priapulid worm in the phylum Scalidophora, contains 5 such proteins (Mcl-1, A1, Bok, Bax, Bcl-2R1, Supporting Data). The lophotrochozoans (*Schmidtea mediterranea*, *S. japonicum*, *S. mansoni*) have multiple Bcl-2-like genes and an apparently tripartite (pro-survival Bcl-2, pro-apoptotic Bcl-2, and BH3-only components) system for apoptosis signalling [55,56] comparable to that in mammals. Similarly, the Lophotrochozoans, *Adineta vaga* (Rotifer, 3 Bcl-2 proteins), *Lingula anatina* (Brachiopoda) [57], *Capitella teleta* [58], *Helobdella robusta* [59] (Annelida) all contain multiple Bcl-2 proteins.

In the Deuterostome clade, like the protostomes, some members have undergone extensive gene loss. The tunicates (Urochordates) present a more complex picture. This group forms a sister group to the chordates [59]. The tunicates *Ciona intestinalis* [60] and *Botryllus schlosseri* [61] contain multi-motif Bcl-2 proteins. On the other hand, the Appendicularian *Oikopleura dioica* [62] from the sister group to all other tunicates [63] has lost all Bcl-2 members. The sister group to chordates Echinodermata (Figure 4a) have multiple Bcl-2 proteins present in their genomes, for example, the genome of echinoderms *Strongylcentrotus purpuratus* [64] may contain as many as 8 Bcl-2 proteins, Crown of thorns star fish, *Acanthaster planci* 7 Bcl-2 proteins, and Sea cucumber *Apostichopus japonicum* 6 Bcl-2 proteins. While the Hemichordate *Saccoglossus kowalevskii* (Bax, Bak, Bcl-2R1L, Mcl-1L) and Cephalochordate *Branchiostoma belcheri* (Bcl-2L1, NR13, Bak, Bok-LB) feature multiple Bcl-2 proteins, none cluster with Mcl-1, Bcl-2A1 or Bcl-2L10, (Figure 4b) and *S. kowalevskii* has no Bak or Bok clustering Bcl-2 protein. Interestingly, many of these organisms have multiple sequences that cluster with Bok. Thus, like the Protostomes, some Deuterostomes have undergone gene loss events that have simplified or removed the Bcl-2 family. Broadly, the Bcl-2 proteins from the basal bilaterians cluster with the mammalian Bcl-2 family members with the exception of Mcl-1/A1/Bcl-B, whilst featuring a gene structure that is maintained.

### 2.4. BH3-Only Proteins

Employing the BH3-only protein sequence from *C. elegans*, EGL-1, and the schistosomes *S. japonicum* and *S. mansoni* as seeds using an orthogonal search strategy, we performed a search for analogues in the branches of protostomes (for example the Lophotrochozoan branch was searched with an Ecdysozoan BH3 sequence, EGL-1). This strategy proved unsuccessful in discovery of BH3-only proteins in the alternate branches of protostomes and suggests that either the BH3-only proteins in these organisms are phylogenetically unrelated or arose independently in convergent evolution, diverged significantly over evolutionary timespans, or are simply absent.

Recognizable BH3-only proteins could not be identified in sponge (*A. queenslandica*) or *Trichoplax* genomes using either the BH3-only protein sequences from *C. elegans* (Egl-1) and *S. japonicum* (sJC) [55] or mammalian BH3-only proteins as seeds. The finding that BH3-only proteins are apparently absent from *T. adhaerens* is consistent with others [26]. Representatives of the Bcl-2 family, including BH3-only proteins, were not identified in ctenophores. The evolutionarily earliest BH3-only proteins identified so far occur in the cnidarian *H. vulgaris* [24] but our search of the related myxozoans, *Thelohanellus kitaue* and *Kudoa iwatai*, did not find identifiable multi-motif Bcl-2 proteins or their BH3-only protein relatives.

**Figure 5:**
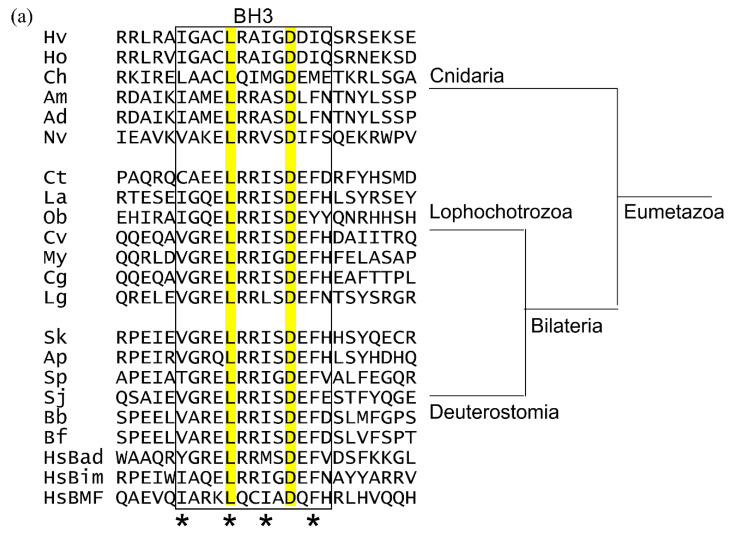

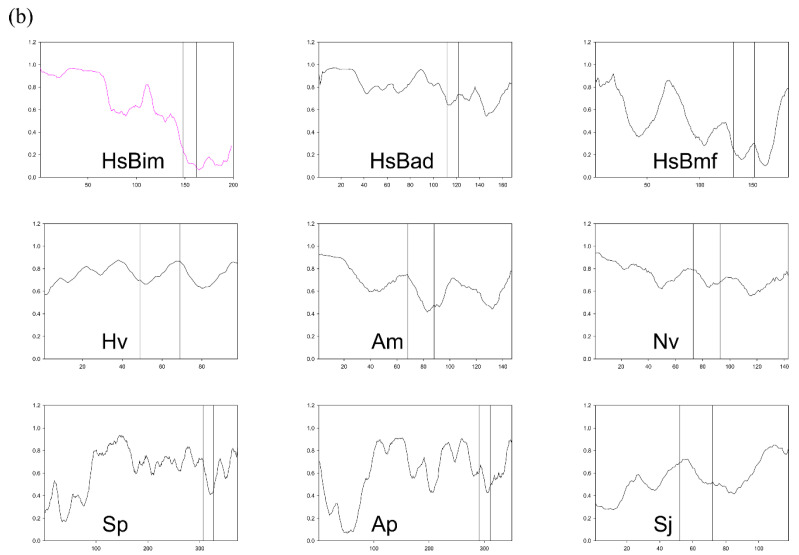
Potential BH3-only proteins from selected early metazoans (**a**) multiple protein sequence alignment of gene structures protein sequences of the BH3 region only are shown. Cnidarian BH3-only genes lack introns. The key conserved leucine and aspartate of the BH3 region are highlighted. (**b**) The disordered nature of BH3-only proteins is conserved. Plots of averaged predicted IDP indices versus sequence position for human and hypothesised Cnidarian BH3-only proteins. Analysis of potential BH3-only proteins and comparison with known BH3-only proteins from humans. Human Bim (HsBim), human Bad (HsBad), human Bmf (HsBmf). The extent of the BH3 region is demarked by the box and the key hydrophobic positions are indicated by *. The IDP indices were calculated by averaging the IDP indices from PONDR (VL-XT, VSL2, VL3), PONDR-Fit and IUPred2A. Abbreviations Cnidarians: Hv, *Hydra vulgaris*; Ho, *Hydra oligactis*; Am, Ch, *Clytia hemisphaerica*; *Acropora millepora*; Ad, *Acropora digitifera*; Nv, *Nematostella vectensis*; Lophochorozoa: Ct, *Capitella teleta*; La, *Lingula anatine*; Ob, *Octopus bimaculoides*; Cv, *Crassostrea virginica*; My, *Mizuhopecten yessoensis*; Cg, *Crassostrea gigas*; Lg, *Lottia gigantea*; Deuterstomia: Sk, *Saccoglossus kowalevskii*; Ap, *Acanthaster planci*; Sp, *Strongylocentrotus purpuratus*; Sj, *Stichopus japonicus*; Bb, *Branchiostoma belcheri*; Bf, *Branchiostoma floridae*; Hs, *Homo sapiens.*

Using the human BH3-only proteins Bim, Bad, and Bmf as seed sequences in a BLAST search of the NCBI database, probable BH3-only proteins were found in Deuterostomes but not Urochordates, and are potentially present in Lophotrochozoa. Potential BH3-only proteins were revealed in Echinoderms using the seed sequences of human Bim, Bad and Bmf (Figure 5a). Possible BH3-only proteins were found in the genomes of the Echinoderms, *S. japonicus*, *S. purpuratus,* and *A. planci*. The *S. japonica* sequence is 118 residues in length but the sequences identified as potential BH3-only proteins in *S. purpuratus* and *A. planci* have much longer sequences than mammalian or cnidarian BH3-only proteins, of 373 residues and 350 residues, respectively. The BH3 region occurs on a single exon in all cases. All three sequences are predicted to be substantially unstructured (Figure 5b), consistent with these proteins as IDPs and ‘hub proteins’. While these proteins fit the pattern of BH3-only proteins, confirmatory experimental evidence is required to place them as Bcl-2 family members.

Homologs of many mammalian BH3-only proteins were identified in fish. A Bad homolog was identified in Actinista (Coelacanths), while Bim and Bmf are absent. Bid does not appear until Chondrichthyes (sharks and rays) or in Holostei (*Lepisosteus oculatus*, spotted garfish [65]) and has clear homologues in *D. rerio*. Bik-like, Puma-like, and Noxa-like sequences could be found in fish, but Hrk appears absent. Bid and other BH3-only proteins appear absent from hagfish (*Eptatretus burgeri*) and lamprey (*Petromyzon marinus*).

Evidence was sought for the intrinsically disordered nature of the BH3-only proteins, a key feature of this class of Bcl-2 protein [66,67], using a computational approach based on averaging the per residue IDP predictors [68] with the programs Predictors of Natural Disorder (PONDR) [69] and IUPred [70] and averaging the per residue IDP parameters. Figure 5b shows the plots of averaged IDP indices; indices greater than 0.5 are indicative of unstructured regions. Potential BH3-only proteins in cnidarians and echinoderms had protein sequences predicted to be substantially disordered consistent with their BH3-only potential (Figure 5b).

### 2.5. Other Genes Associated with Intrinsic Apoptosis

The apoptotic genes downstream of Bcl-2 family members include apical caspase activators such as Apaf-1 and executioner caspases, and are required for cell component destruction of intrinsic apoptosis. In addition to the absence of discernible Bcl-2 proteins in myxozoans *T. kitaue* and *K. iwatai*, Apaf-1 like genes were not found in either genome using the sequences of *H. vulgaris* as a search seed, a finding that is consistent with Panchin et al. [49]. Thus, in both ctenophores and myxozoans, the genes related to MOMP initiated apoptosis appear to have been lost. Other Bilaterians have also lost Bcl-2 family members such as *H. miamia*, and Clade IV nematode species *Meloidogyne*, *Globodera*, and *Ditylenchus* [31]; in the case of *H. miamia,* caspase-7 and caspase-8 genes are present [53].

The lophotrochozoans (*Schmidtea mediterranea*, *S. japonicum*, *S. mansoni*) have an Apaf-1 gene that initiates the caspase cascade [55,56]. However, the presence of Apaf-1 may not necessarily indicate MOMP as an activator of the caspase cascade. Activation of apoptosis in echinoderms (ambulacraria) may not involve cytochrome *c* release from mitochondria, cytochrome *c* is not apparently necessary for Apaf-1 activated apoptosis in the starfish *Asterina pectinifera* [71]. The presence of Apaf-1 therefore may not be definitive for an intrinsic apoptotic mechanism dependent on mitochondrial cytochrome *c* release. Tunicates (Urochordates) lack an obvious Apaf-1 candidate but possess several CED4 like caspases indicating cytochrome *c*-initiated apoptosis either does not occur or is highly modified in these organisms. Figure 4 summarizes the presence of these apoptotic genes.

## 3. Discussion

Remarkably, the key sequence features of the Bcl-2 family, the BH1, BH2, and BH3 motifs have remained stable throughout metazoan history and are clearly visible in the Bcl-2 proteins from the demosponge *G. cydonium*, [23,40], placozoan, *T. adhaerens* [26,30] and cnidarian *H. vulgaris* [25,29], and bilaterians [6] (Figure 1 and Figure 2). Database searches of representative genomes from the five basal metazoan clades porifera, ctenophora, placozoa, cnidaria, and bilateria identified Bcl-2 genes in all clades with the exception of ctenophora. Significantly, our sequence and structure-based database searches did not identify non-Bcl-2 proteins bearing the sequence signature of BH motifs within the same organisms. In contrast to genomes of other basal metazoans, Bcl-2 family proteins were not identified in the genomes of the ctenophores (sea jellies) *M. leidyi* [47] or *P. bachei* [48], or in myxozoans, obligate parasites closely related to cnidarians. Furthermore, database searches reported here and by others [17] did not identify Bcl-2 proteins in pre-metazoans, such as the choanozoans and its sister clade filastereans (e.g., *Monosiga brevicollis* [72] and *Capsapora owczarzaki* [73], a choanaflagellate and a filasterean, respectively) a group of single-celled organisms postulated to be a sister group to the metazoans [74]. Although some viral genomes contain examples of Bcl-2 proteins [75], the Bcl-2 family appears to be a uniquely metazoan set of genes.

Many genomes of basal metazoans contain multiple Bcl-2-like genes that cluster with mammalian Bcl-2 genes (Figure 3) and points to the establishment of complex Bcl-2 initiated signalling early in metazoan evolution (Figure 3 and Figure 4c). For example, porifera, organisms considered the sister group to all metazoans [76,77], contain representatives such as the sponge *A. queenslandica* [28], an organism of approximately 40,122 genes [78] of which 6 are putative multi-motif Bcl-2 genes, including those that cluster with Bak and Bcl-2. In contrast to *A. queenslandica,* the sponge *G. cydonium* bears only two Bcl-2 proteins (BHP1, BHP2) in its genome [40]. The placozoan *T. adhaerens*, an organism with only 11,514 genes [30] and six somatic cell types [79] includes four Bcl-2 homologs [26,30]. 11 Bcl-2 proteins were identified in the 24,450 genes [80] of the cnidarian *H. vulgaris* [24] (Figure 2). While ctenophores do not harbour Bcl-2 family genes, we detected them in the tunicate *C. intestinalis* [60], including Bok, Bax, and Bcl-2 homologues; however, we were unable to detect them in the tunicate *Oikopleura dioica*. Early Bilaterians generally have genes that cluster with the mammalian Bcl-2 family (Figure 4b). The absence of Bcl-2 genes in ctenophores, myxozoans, some tunicates, and certain nematodes [31] suggests Bcl-2 gene loss is not critical to survival, homeostasis, development and multicellularity in these organisms.

The key feature of pro-apoptotic proteins is the BH3-motif and it appears in the Bax/Bak-like proteins of porifera but we were unable to identify clear candidates for BH3-only proteins in either porifera or placozoa. Consistent with Lasi et al. in their investigation of hydra [24], we identified BH3-only candidates in cnidarian genomes (Figure 5). We also observed that BH3-only proteins were not found where the multi-motif Bcl-2 genes have been deleted, such as the myxozoans. In the bilaterians, BH3-only proteins have been identified in some but not all Ecdysozoa (*C. elegans* [81], but not other branches of the nematodes [31] or insects, *D. melanogaster*). The nematodes that lack a Bcl-2 protein also lack a homolog of the BH3-only gene *egl1*. The platyhelminth genomes *Schistosoma japonicum* and *Schistosoma mansoni* bear BH3-only proteins and these Lophotrochozoans mimic the tripartite Bcl-2 function in mammals [55]. Performing sequence searches based on either the schistosome or nematode BH3-only protein sequences in an orthogonal search strategy, we failed to identify homologues in the alternate organisms; this suggests that BH3-only proteins from schistosomes and nematodes are not phylogenetically closely related and may have arisen by convergent evolution in the nematodes. In deuterostomes, we identified potential BH3-only proteins in Echinoderms, Hemichordates (i.e., the Ambulacraria arm of deuterostomes), the Chordates Cephalochordata and vertebrates, but not in Urochordate *O. doica* as expected. Bid and other BH3-only proteins appear absent from hagfish (*E. burgeri*) and lamprey (*P. marinus*); however, several multi-motif Bcl-2 proteins are present in these cases. Using mammalian Bad as a seed, a homologue was identified in Actinista (Coelacanths) while Bim and Bmf are absent. Bim, Bmf and Bid appear in Chondrichthyes (sharks and rays) and these are accompanied by Puma in Holostei (*L. oculata*, garfish). Homologues of Bik-like, Puma-like, and Noxa-like as well as Bim, Bad, Bmf, and Bid are present in the zebrafish *D. rerio*, but Hrk appears absent. In combination, these findings imply the BH3-only proteins arose later in evolution than the multi-motif Bcl-2 family.

Structural comparisons on the *G. cydonium*, *T. adhaerens,* and *H. vulgaris* Bcl-2 proteins with those from higher organisms demonstrate that the 3D structure of Bcl-2 proteins is essentially topologically invariant over evolutionary history (Figure 2). The uniqueness of the Bcl-2 fold is suggestive of a monophyletic origin and derivation from a common ancestor [82], though such an ancestor has yet to be identified. Moreover, the sequence and structure conservation suggest that the Bax–Bcl-2 interaction is preserved from early metazoans. Sequence analysis predicts that the BH3-only proteins are IDPs and is consistent with the known BH3-only proteins [67]. The occurrence of Bim, Bad, and Bmf prior to Bid infers that intrinsic disorder of BH3-only proteins is fundamental to their action. Structural studies indicate that similar to their mammalian counterparts, the BH3-in-groove mechanism is likely to be the basis of Bcl-2 protein action in early metazoans [23,25,26] and the BH3-in-groove interaction mechanism between pro-apoptotic and pro-survival proteins is conserved from sponges to man. Combined, these data point to the presence of a sophisticated network of Bcl-2 protein activity in early metazoans.

The question arises as to when the mechanism of activation of apoptosis by cytochrome *c* occurred [4]. Currently there is little functional data available for the early metazoans. Certain placozoan Bcl-2 homologs localize to mitochondria in mammalian cells and expression of Bax/Bak homologs lead to cytochrome *c* release and caspase 3 activation, the hallmarks of apoptosis [26]. The hydra Bcl-2 protein, HyBcl-2-4, localizes to mitochondria and prevents apoptosis [83] and hydra Bcl-2 proteins interact [24,25]. The structural basis of apoptosis of Bcl-2 mediated apoptosis in placozoan [26] and hydra [25] revealed that they rely on the BH3-in-groove mechanism observed in higher organisms [25] and indicate that mitochondrially signalled apoptosis is possible in placozoans and cnidarians. Experiments on cytosolic extracts from Echinoderms *Strongylocentrotus purpuratus* (purple sea urchin) and *Dendraster excentricus* (sand dollar) indicated caspase activation was induced with cytochrome *c* [56]. However, investigation of the nematodes *Trichuris suis* and *Plectus sambesii* showed their Bcl-2 homologs localized to the MOM but neither interaction with BH3 motifs nor cytochrome *c* release were observed [31]. In contrast, Bak and Bcl-2 homologs appear the basis of cytochrome *c* release and apoptosis in platyhelminths (*S. mediterranea* and *Dugesia dorotocephala*) [56] and potential BH3-only proteins occur in the genomes of platyhelminths *S. japonicum* and *S. mansoni* [55]. Together, the current data from phyla Lophotrochozoa and Echinodermata indicate that in contrast to the Ecdysozoans intrinsic apoptosis occurs via cytochrome *c* triggered MOMP (Figure 4c). While homologs of modulators of apoptosis such as caspases and Bcl-2 family members exist in *T. adhaerens* [4,26], their exact functions are yet to be verified, though structural biochemical and functional studies indicate functional conservation between this basal metazoan [26] with bilaterian intrinsic apoptosis.

## 4. Materials and Methods

Bcl-2 orthologues were sought initially in the OrthoDB database (https://www.orthodb.org) [84] accessed on 29 July 2020. Sequence searches were performed using the NCBI databases and BLAST (https://blast.ncbi.nlm.nih.gov/Blast.cgi) [33] accessed on 29 July 2020 using sequences of known Bcl-2 proteins from human (Bcl-2, Bcl-x_L_, Bcl-w, Mcl-1, A1, Bcl-B, Bax, Bak, Bok, Bim Bad Bmf, Bid, Bik, Puma, Hrk, Noxa), *G. gallus* (Bcl-2, Bcl-2l1, Bcl-2A1, Baxb, NR13, Bok, Ml-1, Bak1) nematode (*C. elegans* CED9, Egl-1), zebrafish (*D. rerio* Bcl-2a, Bcl-2b, Bcl-2l1, Bcl-2l6, Boka, Bokb, Bcl-2l10, Mcl-1a, Mcl-1b, Baxa, Baxb), sponge (*G. cydonium BHP1*, *BHP2*) and *H. vulgaris* [24] as seeds.

Sequence database searches were performed using the following: Ensembl, GeneDB, Broad Institute, Joint Genome Institute (JGI), National Human Genome Research Institute (NHGRI), National Center for Biotechnology Information (NCBI). The OIST Genome browser (http://marinegenomics.oist.jp/gallery/) (accessed on 29 July 2020) database was used for searches of the genomes of the Cnidarians, *M. virulenta*, [46], *A. aurita* [46] using the *H. vulgaris* Bcl-2 protein sequences as seeds. Genomes of Echinoderms: *Strongylcentrotus purpuratus* [64] (NCBI), Crown of thorns star fish, *Acanthaster planci* [85], and *Apostichopus japonicum* were searched using the OIST Genome browser. Tunicate Bcl-2 proteins in *O. dioica* were sought using OikoBase (http://oikoarrays.biology.uiowa.edu/Oiko/) [86] accessed on 29 July 2019 and those present in *B. schlosseri* (http://octopus.obs-vlfr.fr/public/botryllus/blast_botryllus.php) [87] accessed on 29 July 2019 using BLAST searches. Early branching metazoa *Oscarella carmela*, (homoscleromorpha), *Sycon ciliatum* (Calcarea) were obtained from Blast searches of Compagen (http://www.compagen.org) accessed on 29 July 2019.

Hidden Markov Model (HMM) [34,88] searches were performed either on the MPI site https://toolkit.tuebingen.mpg.de (accessed on 29 July 2019) against the standard non-redundant databases 0, 30, 50, and 90% [89] or the EBI https://www.ebi.ac.uk/Tools/hmmer/ [90] (accessed on 29 July 2019) using full-length Bcl-2 proteins. Iterative HMM searches were performed on the EBI site using JACKHMMER using seed sequences of known or predicted Bcl-2 proteins. Sequence alignments were performed in MEGA7 [35] using Muscle with the standard parameters provided. Phylogenetic trees were calculated in MEGA7 [35] with 1000 steps bootstrap, maximum likelihood method. Structural similarity searches of the PDB database were performed with DALI (http://ekhidna2.biocenter.helsinki.fi/dali/) [37] (accessed on 29 July 2019).

Bcl-2 fold and BH regions were confirmed using the NCBI Conserved Domain Database (CDD) https://www.ncbi.nlm.nih.gov/Structure/cdd/wrpsb.cgi [36] accessed on 29 July 2019.

Structure based phylogenetic trees were generated from the structure-based alignment produced in DALI [37].The intrinsically disordered status of the predicted BH3-only proteins were determined using the averaged per residue outputs from the IDP predictors from the Predictor Of Natural Disorder Regions (PONDR) family (PONDR: VLXT, VSL2, VL3) (http://www.pondr.com/) [68] (accessed on 29 July 2019), PONDR-FIT (http://original.disprot.org/pondr-fit.php) [91] (accessed on 29 July 2019) and IUPred (https://iupred2a.elte.hu/) [70] (accessed on 29 July 2019). This method improves the predictive performance of the IDP prediction [92].

## 5. Conclusions

A generally accepted consensus on the phylogeny of metazoans has yet to emerge, in part due to the difficulty in identifying and differentiating orthologous and paralogous proteins [93] (Figure 4a), and this seems particularly true in the Bcl-2 family, where there are multiple members and a complex network of interactions. However, the advent of gene sequencing and structural biology initiatives and searchable databases has made it possible to compare sequences and structures across evolutionary timeframes. While it is unlikely that a single gene family, such as the Bcl-2 family, can resolve the conflicts in evolutionary paths that have arisen in metazoan lineages [94], the conservation of the Bcl-2 family in metazoans makes them a model candidate for aiding the delineation of evolutionary relationships. The question of the ultimate metazoan ancestor, the ‘Ur’ metazoan, remains unresolved, but the Bcl-2 proteins appear early in metazoan evolution. The absence of Bcl-2 genes in some metazoans indicates that mitochondrial activated apoptosis is not an obligate feature of metazoans and the absence of the BH3-only group in the earliest metazoans, but not all metazoans, also indicates they are non-obligatory in intrinsic apoptosis. This finding is consistent with the observation that mitochondria-based apoptosis can be triggered in the absence of BH3-only proteins [95]. Furthermore, knockout of Bak, Bax, and Bok, the key genes in mitochondria-signalled apoptosis in mice, showed this pathway is not strictly necessary, at least for developmental apoptosis [96]. Ecdysozoans appear to have lost most Bcl-2 proteins and in some cases in their entirety [31]. There are clearly differences in Bcl-2 mechanisms, for example, hydra appear to have the mammal-like tripartite apoptosis signalling (pro-survival, pro-apoptotic and BH3-only members) but in placozoans Bak-like protein may substitute for the BH3-only proteins. The nematodes have a much-reduced dependence on their Bcl-2 proteins and like some earlier metazoans have gene loss that has eliminated the family altogether. Many early metazoans have greatly expanded Bcl-2 families when compared to later metazoans and the contraction in the number of Bcl-2 genes higher metazoans corresponds to the advent of adaptive immune responses. Examining the foundations of Bcl-2 regulated apoptosis functional relationships may clarify and deepen the role this family in evolution and disease [97].

## Figures and Tables

**Table 1:** Sequence identity and similarity analysis of Bcl-2 proteins from representative metazoans from early to late evolution. Sequence identities and similarity determined for the structure sequences using the sequence manipulation suite [39] reported as percentage identity/similarity.

Bcl-2 Protein					
taBcl2l4	18.0/30.5				
hvBcl2	16.6/32.0	36.5/50.3			
ceCED9	10.7/25.3	13.5/28.1	10.5/25.4		
drNRZ	15.6/27.5	17.7/26.8	18.6/33.5	10.7/23.0	
hsBcl2	16.0/28.6	24.2/39.8	24.9/40.0	11.8/27.7	17.8/28.8
	gcBHP2	taBcl24	hvBcl2	ceCED9	drNRZ

## Data Availability

All sequences were sourced from publicly funded and accessible databases and are thus freely available.

## Supplementary Materials

The following are available online at https://www.mdpi.com/article/10.3390/ijms23073691/s1.

## References

[1] Green D.R., Llambi F. (2015). Cell Death Signaling. Cold Spring Harb. Perspect. Biol..

[2] Sakamaki K., Imai K., Tomii K., Miller D.J. (2015). Evolutionary analyses of caspase-8 and its paralogs: Deep origins of the apoptotic signaling pathways. Bioessays.

[3] Degterev A., Yuan J. (2008). Expansion and evolution of cell death programmes. Nat. Rev. Mol. Cell. Biol..

[4] Green D.R., Fitzgerald P. (2016). Just So Stories about the Evolution of Apoptosis. Curr. Biol..

[5] Koonin E.V., Aravind L. (2002). Origin and evolution of eukaryotic apoptosis: The bacterial connection. Cell Death Differ..

[6] Banjara S., Suraweera C.D., Hinds M.G., Kvansakul M. (2020). The Bcl-2 Family: Ancient Origins, Conserved Structures, and Divergent Mechanisms. Biomolecules.

[7] Kroemer G. (1997). Mitochondrial implication in apoptosis. Towards an endosymbiont hypothesis of apoptosis evolution. Cell Death Differ..

[8] Kvansakul M., Hinds M.G. (2015). The Bcl-2 family: Structures, interactions and targets for drug discovery. Apoptosis.

[9] Kvansakul M., Hinds M.G. (2014). The structural biology of BH3-only proteins. Methods Enzymol..

[10] Chen L., Willis S.N., Wei A., Smith B.J., Fletcher J.I., Hinds M.G., Colman P.M., Day C.L., Adams J.M., Huang D.C. (2005). Differential targeting of prosurvival Bcl-2 proteins by their BH3-only ligands allows complementary apoptotic function. Mol. Cell.

[11] Kuwana T., Bouchier-Hayes L., Chipuk J.E., Bonzon C., Sullivan B.A., Green D.R., Newmeyer D.D. (2005). BH3 domains of BH3-only proteins differentially regulate Bax-mediated mitochondrial membrane permeabilization both directly and indirectly. Mol. Cell.

[12] Rautureau G.J., Yabal M., Yang H., Huang D.C., Kvansakul M., Hinds M.G. (2012). The restricted binding repertoire of Bcl-B leaves Bim as the universal BH3-only prosurvival Bcl-2 protein antagonist. Cell Death Dis..

[13] Shamas-Din A., Kale J., Leber B., Andrews D.W. (2013). Mechanisms of action of bcl-2 family proteins. Cold Spring Harb. Perspect. Biol..

[14] Wilson-Annan J., O’Reilly L.A., Crawford S.A., Hausmann G., Beaumont J.G., Parma L.P., Chen L., Lackmann M., Lithgow T., Hinds M.G. (2003). Proapoptotic BH3-only proteins trigger membrane integration of prosurvival Bcl-w and neutralize its activity. J. Cell Biol..

[15] Popgeorgiev N., Jabbour L., Gillet G. (2018). Subcellular Localization and Dynamics of the Bcl-2 Family of Proteins. Front. Cell Dev. Biol..

[16] Gao S., Fu W., Durrenberger M., De Geyter C., Zhang H. (2005). Membrane translocation and oligomerization of hBok are triggered in response to apoptotic stimuli and Bnip3. Cell Mol. Life Sci..

[17] Aouacheria A., Le Goff E., Godefroy N., Baghdiguian S., Pontarotti P. (2016). Evolution of the BCL-2-Regulated Apoptotic Pathway. Evolutionary Biology: Convergent Evolution, Evolution of Complex Traits, Concepts and Methods.

[18] Zmasek C.M., Godzik A. (2013). Evolution of the animal apoptosis network. Cold Spring Harb. Perspect. Biol..

[19] Metzstein M.M., Stanfield G.M., Horvitz H.R. (1998). Genetics of programmed cell death in *C*. *elegans*: Past, present and future. Trends Genet..

[20] Glasauer S.M., Neuhauss S.C. (2014). Whole-genome duplication in teleost fishes and its evolutionary consequences. Mol. Genet. Genom..

[21] Kratz E., Eimon P.M., Mukhyala K., Stern H., Zha J., Strasser A., Hart R., Ashkenazi A. (2006). Functional characterization of the Bcl-2 gene family in the zebrafish. Cell Death Differ..

[22] Suraweera C.D., Caria S., Jarva M., Hinds M.G., Kvansakul M. (2018). A structural investigation of NRZ mediated apoptosis regulation in zebrafish. Cell Death Dis..

[23] Caria S., Hinds M.G., Kvansakul M. (2017). Structural insight into an evolutionarily ancient programmed cell death regulator—the crystal structure of marine sponge BHP2 bound to LB-Bak-2. Cell Death Dis..

[24] Lasi M., Pauly B., Schmidt N., Cikala M., Stiening B., Kasbauer T., Zenner G., Popp T., Wagner A., Knapp R.T. (2010). The molecular cell death machinery in the simple cnidarian Hydra includes an expanded caspase family and pro- and anti-apoptotic Bcl-2 proteins. Cell Res..

[25] Banjara S., Sa J.D., Hinds M.G., Kvansakul M. (2020). The structural basis of Bcl-2 mediated cell death regulation in hydra. Biochem. J..

[26] Popgeorgiev N., Sa J.D., Jabbour L., Banjara S., Nguyen T.T.M., Akhavan E.S.A., Gadet R., Ralchev N., Manon S., Hinds M.G. (2020). Ancient and conserved functional interplay between Bcl-2 family proteins in the mitochondrial pathway of apoptosis. Sci. Adv..

[27] dos Reis M., Thawornwattana Y., Angelis K., Telford M.J., Donoghue P.C., Yang Z. (2015). Uncertainty in the Timing of Origin of Animals and the Limits of Precision in Molecular Timescales. Curr. Biol..

[28] Srivastava M., Simakov O., Chapman J., Fahey B., Gauthier M.E., Mitros T., Richards G.S., Conaco C., Dacre M., Hellsten U. (2010). The Amphimedon queenslandica genome and the evolution of animal complexity. Nature.

[29] Chapman J.A., Kirkness E.F., Simakov O., Hampson S.E., Mitros T., Weinmaier T., Rattei T., Balasubramanian P.G., Borman J., Busam D. (2010). The dynamic genome of Hydra. Nature.

[30] Srivastava M., Begovic E., Chapman J., Putnam N.H., Hellsten U., Kawashima T., Kuo A., Mitros T., Salamov A., Carpenter M.L. (2008). The Trichoplax genome and the nature of placozoans. Nature.

[31] Young N.D., Harris T.J., Evangelista M., Tran S., Wouters M.A., Soares da Costa T.P., Kershaw N.J., Gasser R.B., Smith B.J., Lee E.F. (2020). Diversity in the intrinsic apoptosis pathway of nematodes. Commun. Biol..

[32] King N., Rokas A. (2017). Embracing Uncertainty in Reconstructing Early Animal Evolution. Curr. Biol..

[33] Altschul S.F., Madden T.L., Schaffer A.A., Zhang J., Zhang Z., Miller W., Lipman D.J. (1997). Gapped BLAST and PSI-BLAST: A new generation of protein database search programs. Nucleic Acids Res.

[34] Eddy S.R. (2011). Accelerated Profile HMM Searches. PLoS Comput. Biol..

[35] Kumar S., Stecher G., Tamura K. (2016). MEGA7: Molecular Evolutionary Genetics Analysis Version 7.0 for Bigger Datasets. Mol. Biol. Evol..

[36] Marchler-Bauer A., Bo Y., Han L., He J., Lanczycki C.J., Lu S., Chitsaz F., Derbyshire M.K., Geer R.C., Gonzales N.R. (2017). CDD/SPARCLE: Functional classification of proteins via subfamily domain architectures. Nucleic Acids Res..

[37] Holm L., Laakso L.M. (2016). Dali server update. Nucleic Acids Res..

[38] Holm L. (2020). DALI and the persistence of protein shape. Protein Sci..

[39] Stothard P. (2000). The sequence manipulation suite: JavaScript programs for analyzing and formatting protein and DNA sequences. Biotechniques.

[40] Wiens M., Diehl-Seifert B., Muller W.E. (2001). Sponge Bcl-2 homologous protein (BHP2-GC) confers distinct stress resistance to human HEK-293 cells. Cell Death Differ..

[41] Wiens M., Belikov S.I., Kaluzhnaya O.V., Schroder H.C., Hamer B., Perovic-Ottstadt S., Borejko A., Luthringer B., Muller I.M., Muller W.E. (2006). Axial (apical-basal) expression of pro-apoptotic and pro-survival genes in the lake baikal demosponge *Lubomirskia baicalensis*. DNA Cell Biol..

[42] Kortschak R.D., Samuel G., Saint R., Miller D.J. (2003). EST analysis of the cnidarian *Acropora millepora* reveals extensive gene loss and rapid sequence divergence in the model invertebrates. Curr. Biol..

[43] Shinzato C., Shoguchi E., Kawashima T., Hamada M., Hisata K., Tanaka M., Fujie M., Fujiwara M., Koyanagi R., Ikuta T. (2011). Using the *Acropora digitifera* genome to understand coral responses to environmental change. Nature.

[44] Putnam N.H., Srivastava M., Hellsten U., Dirks B., Chapman J., Salamov A., Terry A., Shapiro H., Lindquist E., Kapitonov V.V. (2007). Sea anemone genome reveals ancestral eumetazoan gene repertoire and genomic organization. Science.

[45] Gold D.A., Katsuki T., Li Y., Yan X., Regulski M., Ibberson D., Holstein T., Steele R.E., Jacobs D.K., Greenspan R.J. (2019). The genome of the jellyfish Aurelia and the evolution of animal complexity. Nat. Ecol. Evol..

[46] Khalturin K., Shinzato C., Khalturina M., Hamada M., Fujie M., Koyanagi R., Kanda M., Goto H., Anton-Erxleben F., Toyokawa M. (2019). Medusozoan genomes inform the evolution of the jellyfish body plan. Nat. Ecol. Evol..

[47] Ryan J.F., Pang K., Schnitzler C.E., Nguyen A.D., Moreland R.T., Simmons D.K., Koch B.J., Francis W.R., Havlak P., Program N.C.S. (2013). The genome of the ctenophore *Mnemiopsis leidyi* and its implications for cell type evolution. Science.

[48] Moroz L.L., Kocot K.M., Citarella M.R., Dosung S., Norekian T.P., Povolotskaya I.S., Grigorenko A.P., Dailey C., Berezikov E., Buckley K.M. (2014). The ctenophore genome and the evolutionary origins of neural systems. Nature.

[49] Panchin A.Y., Aleoshin V.V., Panchin Y.V. (2019). From tumors to species: A SCANDAL hypothesis. Biol. Direct..

[50] Rautureau G.J., Day C.L., Hinds M.G. (2010). The structure of Boo/Diva reveals a divergent Bcl-2 protein. Proteins.

[51] Moya A., Sakamaki K., Mason B.M., Huisman L., Foret S., Weiss Y., Bull T.E., Tomii K., Imai K., Hayward D.C. (2016). Functional conservation of the apoptotic machinery from coral to man: The diverse and complex Bcl-2 and caspase repertoires of Acropora millepora. BMC Genom..

[52] Aouacheria A., Brunet F., Gouy M. (2005). Phylogenomics of life-or-death switches in multicellular animals: Bcl-2, BH3-Only, and BNip families of apoptotic regulators. Mol. Biol. Evol..

[53] Gehrke A.R., Neverett E., Luo Y.J., Brandt A., Ricci L., Hulett R.E., Gompers A., Ruby J.G., Rokhsar D.S., Reddien P.W. (2019). Acoel genome reveals the regulatory landscape of whole-body regeneration. Science.

[54] Telford M.J., Budd G.E., Philippe H. (2015). Phylogenomic Insights into Animal Evolution. Curr. Biol..

[55] Lee E.F., Clarke O.B., Evangelista M., Feng Z., Speed T.P., Tchoubrieva E.B., Strasser A., Kalinna B.H., Colman P.M., Fairlie W.D. (2011). Discovery and molecular characterization of a Bcl-2-regulated cell death pathway in schistosomes. Proc. Natl. Acad. Sci. USA.

[56] Bender C.E., Fitzgerald P., Tait S.W., Llambi F., McStay G.P., Tupper D.O., Pellettieri J., Sanchez Alvarado A., Salvesen G.S., Green D.R. (2012). Mitochondrial pathway of apoptosis is ancestral in metazoans. Proc. Natl. Acad. Sci. USA.

[57] Luo Y.J., Takeuchi T., Koyanagi R., Yamada L., Kanda M., Khalturina M., Fujie M., Yamasaki S.I., Endo K., Satoh N. (2015). The Lingula genome provides insights into brachiopod evolution and the origin of phosphate biomineralization. Nat. Commun..

[58] Simakov O., Marletaz F., Cho S.J., Edsinger-Gonzales E., Havlak P., Hellsten U., Kuo D.H., Larsson T., Lv J., Arendt D. (2013). Insights into bilaterian evolution from three spiralian genomes. Nature.

[59] Delsuc F., Brinkmann H., Chourrout D., Philippe H. (2006). Tunicates and not cephalochordates are the closest living relatives of vertebrates. Nature.

[60] Dehal P., Satou Y., Campbell R.K., Chapman J., Degnan B., De Tomaso A., Davidson B., Di Gregorio A., Gelpke M., Goodstein D.M. (2002). The draft genome of Ciona intestinalis: Insights into chordate and vertebrate origins. Science.

[61] Voskoboynik A., Neff N.F., Sahoo D., Newman A.M., Pushkarev D., Koh W., Passarelli B., Fan H.C., Mantalas G.L., Palmeri K.J. (2013). The genome sequence of the colonial chordate, Botryllus schlosseri. eLife.

[62] Seo H.C., Kube M., Edvardsen R.B., Jensen M.F., Beck A., Spriet E., Gorsky G., Thompson E.M., Lehrach H., Reinhardt R. (2001). Miniature genome in the marine chordate *Oikopleura dioica*. Science.

[63] Delsuc F., Philippe H., Tsagkogeorga G., Simion P., Tilak M.K., Turon X., Lopez-Legentil S., Piette J., Lemaire P., Douzery E.J.P. (2018). A phylogenomic framework and timescale for comparative studies of tunicates. BMC Biol..

[64] Sea Urchin Genome Sequencing C., Sodergren E., Weinstock G.M., Davidson E.H., Cameron R.A., Gibbs R.A., Angerer R.C., Angerer L.M., Arnone M.I., Burgess D.R. (2006). The genome of the sea urchin *Strongylocentrotus purpuratus*. Science.

[65] Braasch I., Gehrke A.R., Smith J.J., Kawasaki K., Manousaki T., Pasquier J., Amores A., Desvignes T., Batzel P., Catchen J. (2016). The spotted gar genome illuminates vertebrate evolution and facilitates human-teleost comparisons. Nat. Genet..

[66] Rautureau G.J., Day C.L., Hinds M.G. (2010). Intrinsically disordered proteins in Bcl-2 regulated apoptosis. Int. J. Mol. Sci..

[67] Hinds M.G., Smits C., Fredericks-Short R., Risk J.M., Bailey M., Huang D.C., Day C.L. (2007). Bim, Bad and Bmf: Intrinsically unstructured BH3-only proteins that undergo a localized conformational change upon binding to prosurvival Bcl-2 targets. Cell Death Differ..

[68] Huang F., Oldfield C.J., Xue B., Hsu W.L., Meng J., Liu X., Shen L., Romero P., Uversky V.N., Dunker A. (2014). Improving protein order-disorder classification using charge-hydropathy plots. BMC Bioinform..

[69] Obradovic Z., Peng K., Vucetic S., Radivojac P., Brown C.J., Dunker A.K. (2003). Predicting intrinsic disorder from amino acid sequence. Proteins.

[70] Meszaros B., Erdos G., Dosztanyi Z. (2018). IUPred2A: Context-dependent prediction of protein disorder as a function of redox state and protein binding. Nucleic Acids Res..

[71] Tamura R., Takada M., Sakaue M., Yoshida A., Ohi S., Hirano K., Hayakawa T., Hirohashi N., Yura K., Chiba K. (2018). Starfish Apaf-1 activates effector caspase-3/9 upon apoptosis of aged eggs. Sci. Rep..

[72] King N., Westbrook M.J., Young S.L., Kuo A., Abedin M., Chapman J., Fairclough S., Hellsten U., Isogai Y., Letunic I. (2008). The genome of the choanoflagellate *Monosiga brevicollis* and the origin of metazoans. Nature.

[73] Suga H., Chen Z., de Mendoza A., Sebe-Pedros A., Brown M.W., Kramer E., Carr M., Kerner P., Vervoort M., Sanchez-Pons N. (2013). The Capsaspora genome reveals a complex unicellular prehistory of animals. Nat. Commun..

[74] Carr M., Leadbeater B.S., Hassan R., Nelson M., Baldauf S.L. (2008). Molecular phylogeny of choanoflagellates, the sister group to Metazoa. Proc. Natl. Acad. Sci. USA.

[75] Kvansakul M., Caria S., Hinds M.G. (2017). The Bcl-2 Family in Host-Virus Interactions. Viruses.

[76] Feuda R., Dohrmann M., Pett W., Philippe H., Rota-Stabelli O., Lartillot N., Worheide G., Pisani D. (2017). Improved Modeling of Compositional Heterogeneity Supports Sponges as Sister to All Other Animals. Curr. Biol..

[77] Simion P., Philippe H., Baurain D., Jager M., Richter D.J., Di Franco A., Roure B., Satoh N., Queinnec E., Ereskovsky A. (2017). A Large and Consistent Phylogenomic Dataset Supports Sponges as the Sister Group to All Other Animals. Curr. Biol..

[78] Fernandez-Valverde S.L., Calcino A.D., Degnan B.M. (2015). Deep developmental transcriptome sequencing uncovers numerous new genes and enhances gene annotation in the sponge Amphimedon queenslandica. BMC Genom..

[79] Smith C.L., Varoqueaux F., Kittelmann M., Azzam R.N., Cooper B., Winters C.A., Eitel M., Fasshauer D., Reese T.S. (2014). Novel cell types, neurosecretory cells, and body plan of the early-diverging metazoan Trichoplax adhaerens. Curr. Biol..

[80] Wenger Y., Galliot B. (2013). RNAseq versus genome-predicted transcriptomes: A large population of novel transcripts identified in an Illumina-454 Hydra transcriptome. BMC Genom..

[81] Conradt B., Horvitz H.R. (1998). The C. elegans protein EGL-1 is required for programmed cell death and interacts with the Bcl-2-like protein CED-9. Cell.

[82] Koonin E.V., Wolf Y.I., Karev G.P. (2002). The structure of the protein universe and genome evolution. Nature.

[83] Motamedi M., Lindenthal L., Wagner A., Kemper M., Moneer J., Steichele M., Klimovich A., Wittlieb J., Jenewein M., Böttger A. (2019). Apoptosis in Hydra: function of HyBcl-2 like 4 and proteins of the transmembrane BAX inhibitor motif (TMBIM) containing family. Int. J. Dev. Biol..

[84] Kriventseva E.V., Kuznetsov D., Tegenfeldt F., Manni M., Dias R., Simao F.A., Zdobnov E.M. (2019). OrthoDB v10: Sampling the diversity of animal, plant, fungal, protist, bacterial and viral genomes for evolutionary and functional annotations of orthologs. Nucleic Acids Res..

[85] Hall M.R., Kocot K.M., Baughman K.W., Fernandez-Valverde S.L., Gauthier M.E.A., Hatleberg W.L., Krishnan A., McDougall C., Motti C.A., Shoguchi E. (2017). The crown-of-thorns starfish genome as a guide for biocontrol of this coral reef pest. Nature.

[86] Danks G., Campsteijn C., Parida M., Butcher S., Doddapaneni H., Fu B., Petrin R., Metpally R., Lenhard B., Wincker P. (2013). OikoBase: A genomics and developmental transcriptomics resource for the urochordate *Oikopleura dioica*. Nucleic Acids Res..

[87] Rodriguez D., Sanders E.N., Farell K., Langenbacher A.D., Taketa D.A., Hopper M.R., Kennedy M., Gracey A., De Tomaso A.W. (2014). Analysis of the basal chordate *Botryllus schlosseri* reveals a set of genes associated with fertility. BMC Genom..

[88] Remmert M., Biegert A., Hauser A., Soding J. (2011). HHblits: Lightning-fast iterative protein sequence searching by HMM-HMM alignment. Nat. Methods.

[89] Zimmermann L., Stephens A., Nam S.Z., Rau D., Kubler J., Lozajic M., Gabler F., Soding J., Lupas A.N., Alva V. (2018). A Completely Reimplemented MPI Bioinformatics Toolkit with a New HHpred Server at its Core. J. Mol. Biol..

[90] Potter S.C., Luciani A., Eddy S.R., Park Y., Lopez R., Finn R.D. (2018). HMMER web server: 2018 update. Nucleic Acids Res..

[91] Xue B., Dunbrack R.L., Williams R.W., Dunker A.K., Uversky V.N. (2010). PONDR-FIT: A meta-predictor of intrinsically disordered amino acids. Biochim. Biophys. Acta.

[92] Santamaria N., Alhothali M., Alfonso M.H., Breydo L., Uversky V.N. (2017). Intrinsic disorder in proteins involved in amyotrophic lateral sclerosis. Cell Mol. Life Sci..

[93] Pett W., Adamski M., Adamska M., Francis W.R., Eitel M., Pisani D., Worheide G. (2019). The Role of Homology and Orthology in the Phylogenomic Analysis of Metazoan Gene Content. Mol. Biol. Evol..

[94] Nosenko T., Schreiber F., Adamska M., Adamski M., Eitel M., Hammel J., Maldonado M., Muller W.E., Nickel M., Schierwater B. (2013). Deep metazoan phylogeny: When different genes tell different stories. Mol. Phylogenet. Evol..

[95] Huang K., O’Neill K.L., Li J., Zhou W., Han N., Pang X., Wu W., Struble L., Borgstahl G., Liu Z. (2019). BH3-only proteins target BCL-xL/MCL-1, not BAX/BAK, to initiate apoptosis. Cell Res..

[96] Ke F.F.S., Vanyai H.K., Cowan A.D., Delbridge A.R.D., Whitehead L., Grabow S., Czabotar P.E., Voss A.K., Strasser A. (2018). Embryogenesis and Adult Life in the Absence of Intrinsic Apoptosis Effectors BAX, BAK, and BOK. Cell.

[97] Soderquist R.S., Crawford L., Liu E., Lu M., Agarwal A., Anderson G.R., Lin K.H., Winter P.S., Cakir M., Wood K.C. (2018). Systematic mapping of Bcl-2 gene dependencies in cancer reveals molecular determinants of BH3 mimetic sensitivity. Nat. Commun..

